# Measuring facial identity and emotion integration using the redundancy gain paradigm

**DOI:** 10.3758/s13414-018-1603-y

**Published:** 2018-10-08

**Authors:** Leia Vrancken, Elke Vermeulen, Filip Germeys, Karl Verfaillie

**Affiliations:** 1 Brain and Cognition, KU Leuven, Tiensestraat 102, 3000 Leuven, Belgium; 2 Research Centre for Work and Organisation Studies, KU Leuven, Brussels, Belgium

**Keywords:** Face perception, Divided attention, Redundancy gain, Identity/emotion integration

## Abstract

Early theories on face perception posit that invariant (i.e., identity) and changeable (i.e., expression) facial aspects are processed separately. However, many researchers have countered the hypothesis of parallel processes with findings of interactions between identity and emotion perception. The majority of tasks measuring interactions between identity and emotion employ a selective attention design, in which participants are instructed to attend to one dimension (e.g., identity) while the other dimension varies orthogonally (e.g., emotion), but is task irrelevant. Recently, a divided attention design (i.e., the redundancy gain paradigm) in which both identity and emotion are task relevant was employed to assess the interaction between identity and emotion. A redundancy gain is calculated by a drop in reaction time in trials in which a target from both dimensions is present in the stimulus face (e.g., “sad Person A”), compared with trials with only a single target present (e.g., “sad” or “Person A”). Redundancy gains are hypothesized to point to an interactive activation of both dimensions, and as such, could complement designs adopting a selective attention task. The initial aim of the current study was to reproduce the earlier findings with this paradigm on identity and emotion perception (Yankouskaya, Booth, & Humphreys, *Attention, Perception, & Psychophysics*, *74*(8), 1692–1711, 2012), but our study failed to replicate the results. In a series of subtasks, multiple aspects of the design were manipulated separately in our goal to shed light on the factors that influence the redundancy gain effect in faces. A redundancy gain was eventually obtained after controlling for contingencies and stimulus presentation time.

Early theories on face perception arguing that invariant (i.e., identity) versus changeable (i.e., expression) aspects of the face are processed in parallel (Bruce & Young, 1986; Haxby, Hoffman, & Gobbini, 2000) have received confirmation from several lines of research (Calder, Burton, Miller, Young, & Akamatsu, 2001; Calder, Young, Keane, & Dean, 2000; G. W. Humphreys, Donnelly, & Riddoch, 1993; Winston, Henson, Fine-Goulden, & Dolan, 2004). However, many other studies have refuted the face perception model with separate processing of identity and emotion, and point to interactive processing (Baudouin, Martin, Tiberghien, Verlut, & Franck, 2002; Fisher, Towler, & Eimer, 2016; Ganel & Goshen-Gottstein, 2004; Godard, Baudouin, Bonnet, & Fiori, 2013; Kaufmann & Schweinberger, 2004; Levy & Bentin, 2008; Schweinberger & Soukup, 1998; Wang, Fu, Johnston, & Yan, 2013; Yankouskaya et al., 2012), a common visual representation in the neural face network (Blau, Maurer, Tottenham, & McCandliss, 2007; Fox, Moon, Iaria, & Barton, 2009; Ganel, Valyear, Goshen-Gottstein, & Goodale, 2005; Hinojosa, Mercado, & Carretié, 2015; Rhodes et al., 2015), and a facilitating effect of task-irrelevant emotion on face-discrimination training (Lorenzino & Caudek, 2015), thus evolving to new models in which identity and emotion are not completely dissociable, but are processed interactively to some extent (Bernstein & Yovel, 2015; Calder & Young, 2005). The majority of researchers assessing the interaction between identity and emotion employ a selective attention design (e.g., the Garner interference design), in which participants are instructed to attend to one dimension (e.g., identity) while the other dimension varies orthogonally (e.g., emotion), but is task irrelevant (Fisher et al., 2016; Ganel & Goshen-Gottstein, 2004; Schweinberger, Burton, & Kelly, 1999; Schweinberger & Soukup, 1998). In the current paper, we share the latter point of view that posits interactive processing between identity and emotion, but we argue that the operationalization of the emotion–identity interaction is not optimal in a Garner interference task, as human observers simultaneously perceive multiple sources of information in faces in daily life, and are never asked to ignore information from one source. As such, we are convinced that having to monitor multiple sources of information simultaneously during the task (e.g., identity and emotion), here described as divided attention, resembles true face perception far more than selective attention to one source. Indeed, the reverse is actually true: The presence of certain emotions differentially affects the appraisal of an encounter with a given person. For example, when at work, the behavior, thoughts, and intentions of a human observer are quite different when being approached by a coworker expressing an angry emotion compared with a happy emotion.

Recently, some researchers extended the findings on interactive processing of emotion and identity with a divided-attention task (Yankouskaya et al., 2012), defined as the simultaneous monitoring of information along two different dimensions (i.e., identity and emotion). The primary goal of the current paper was to replicate the work by Yankouskaya et al. (2012), adopting a divided-attention task known as the redundancy gain paradigm. The theoretical framework of the paradigm centers around the question whether two sources of information coactivate or not. For example, in a study by Mordkoff and Miller (1993) participants were instructed to respond “target present” whenever a display would show a green figure (green as target “color”), or the letter *X* (*X* as target “shape”). Results showed significantly faster reaction times on trials in which both these targets were present (i.e., a green *X*), hence a *redundant* trial, compared with trials with only one target combined with a nontarget (e.g., a green letter *Y*, or a red-colored *X*). It is then hypothesized that faster reaction times on redundant trials could be the result of two different processes: An independent race between the two dimensions (e.g., color and shape; Raab, 1962), or due to a coactivation (Miller, 1982).

The independent race model posits that faster reaction times result from the separate activation of two information sources, with the faster source in the redundant trial winning the *race* when the participant is instructed to make the decision “target present.” As such, the two sources are processed independently, and on average the winner of the race on redundant trials will be faster than the time needed to make a response on single trials, where only one target is presented. The coactivation model, on the other hand, states that the activation of both sources is combined to generate the decision “target present.” The faster reaction time on redundant trials is the result of two sources building together to reach a single criterion and are thus generally faster than reaction times on single-target trials. Hence, both models support the hypothesis of faster reaction times on redundant trials, but a dissociation can be made following the inequality, proposed by Miller (1982), which must be satisfied if the independent race model holds to be true, but not if results fit a coactivation model:

$$ P\left(\mathrm{RT}<t|\ \mathrm{IE}\right)\le P\left(\mathrm{RT}<\mathrm{t}|\mathrm{I}\right)+P\left(\mathrm{RT}<t|\mathrm{E}\right). $$

This inequality states that the probability of a response RT before a given time *t* on redundant trials (IE) must be equal or less to the combined probabilities of a response before time *t* on the single-target trials (I and E). In other words, the fastest possible response on redundant trials cannot exceed the fastest possible response on the single-target trials. If this is true, the results fit an independent-race model. On the other hand, the inequality must not be satisfied in a coactivation model, since activation from the two sources on redundant trials feed into a common pool, and as such a decision can be reached faster than when only one source is available.

When specifically applied to the perception of identity and emotion in faces, Yankouskaya et al. (2012) found a drop in reaction-time on trials in which a target from both dimensions was present in the stimulus face (e.g., “sad Person A”) compared with trials with only a single target present (e.g., “sad” or “Person A”), thus yielding the conclusion of coactive processing of identity and emotion. This redundancy gain for identity and emotion was replicated multiple times using different identities and emotions, and across different populations (Yankouskaya et al., 2012; Yankouskaya, Humphreys, & Rotshtein, 2014a, 2014b; Yankouskaya, Rotshtein, & Humphreys, 2014). As such, these authors were the first to provide evidence for the interaction between identity and emotion adopting a divided-attention task.

After we conducted a first experiment to replicate the findings from Yankouskaya et al. (2012, Experiment 1), the aim of the present study was to employ the redundancy gain paradigm in a population with congenital prosopagnosia (CP), a face-perception disorder characterized by a deficit in extracting identity information from the face and believed to be present from birth without any (known) straightforward neurological lesions (Cook & Biotti, 2016; Susilo & Duchaine, 2013). Whereas an identity-perception deficit is a central and primary symptom for CP, it is often reported that a clear dissociation exists in the ability of people with CP to recognize identity on the one hand, versus emotion on the other hand, with spared perception for the latter (Duchaine, Parker, & Nakayama, 2003; Humphreys, Avidan, & Behrmann, 2007; Nunn, Postma, & Pearson, 2001; Palermo et al., 2011; but see Biotti & Cook, 2016). In contrast, to our knowledge, the question how, or whether, people suffering from CP integrate multiple sources of face information remains largely unanswered to this day (but see Huis in ’t Veld, Van den Stock, & de Gelder, 2012). However, the finding of coactive processing was not replicated in Experiment 1, and the initial goal of the present study, to assess redundancy gains in CP, was changed to investigate the factors that could affect the redundancy gain paradigm. We manipulated different components of the task in a series of substudies, in order to shed light on the factors that potentially influence the effect. We initially conducted the redundancy-gain paradigm without feedback during the practice trials (Experiment 1) to discourage the matching between pictorial features and the feedback provided (since only six original stimulus faces were presented), but added feedback during Experiment 2 (for a further elaboration on feedback, see the Discussion section after Experiment 1). In Experiment 3, we slightly changed the stimulus set, and in Experiment 4, possible response-stimulus contingencies were removed. Because none of these manipulations proved successful in eliciting a redundancy gain, a perfect replication of Yankouskaya et al. (2012) was conducted for Experiment 5. A redundancy gain was apparent, which was replicated in Experiment 6 with a shorter stimulus-presentation time. Although the redundancy gain was not apparent in Experiments 1–4, all manipulations provided interesting insights into the underlying mechanisms of the redundancy gain paradigm, and a theoretical discussion on these manipulations is thus provided in the Discussion sections after the experiments.

## Experiment 1

### Method

#### Participants

Twenty psychology students participated in the first experiment (12 female, eight male, range: 17–51 years, *M* = 22.9, *SD* = 7.1). The procedure prior to the study was identical in all experiments. Participants were asked to read the onscreen instructions carefully and subsequently sign written informed consents, in conformation to the Medical Ethics Committee of the KU Leuven and the ethical standards laid down in the 1964 Declaration of Helsinki.

#### Stimuli

Stimulus sets in Experiment 1 and all following experiments were obtained from the NimStim face database (Tottenham et al., 2009). Because Yankouskaya et al. (2012) conducted two experiments to determine which pairs of stimuli were on a comparable level of processing speed, we selected the same stimulus faces from the data set to be used in the current studies. Selection of stimuli seems to be important when assessing the interaction between dimensions. To illustrate, in the Garner interference task (i.e., selective attention design in which participants are instructed to categorize objects or faces along one dimension, while ignoring the other dimension), asymmetrical dependencies are believed to be the result of different degrees of discriminability of identity and emotion (Ganel & Goshen-Gottstein, 2004; Wang et al., 2013; but see Schweinberger et al., 1999). Whenever stimuli according to the relevant Dimension A are relatively more difficult to discriminate compared with nonrelevant Dimension B, it is hypothesized that B serves as a reference to help discriminate along dimension A, but not the other way around, resulting in asymmetrical effects (i.e., an interference from B to A, but not from A to B). However, when the efficiency to process the stimuli is low according to the two dimensions, both will be attended and processed (Wang et al., 2013). Applied to the current design, it is possible that when the target expression is not easily detected (i.e., low discriminability) but the target identity is, participants will focus their attention mainly on the identity target to solve the task (i.e., use identity information as a reference), even when they are explicitly instructed to attend both targets equally. For this reason, we chose to select the same stimuli for the current study as Yankouskaya et al. (2012), since their prestudy tasks were meant to rule out such relative differences in discriminability.

**Fig. 1:**
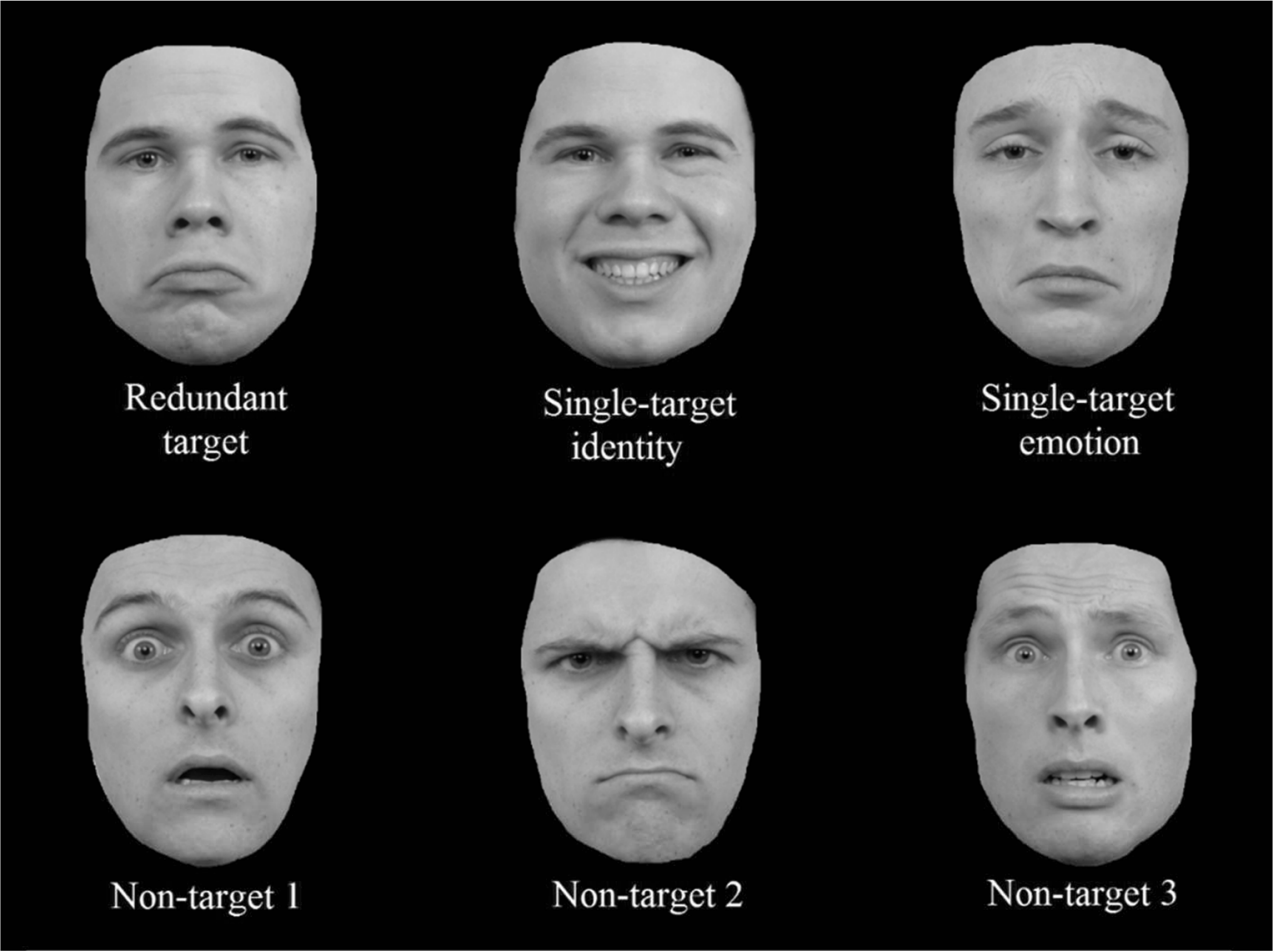
Example stimuli as used in Experiments 1 and 2, taken from the Radboud face database (Langner et al., 2010). The target stimuli consisted of Person A expressing a sad emotion (redundant target), Person A with a happy expression (single-target identity), and Person B with a sad expression (single-target emotion). The nontarget stimuli consisted of Person C expressing fear (Nontarget 1), Person C expressing anger (Nontarget 2), and Person D expressing fear (Nontarget 3). Please note that the faces depicted here were not the faces used in the actual study, but are taken from the Radboud face database to serve as an example, as copyright restrictions prevented us from using the actual stimulus faces

The three target stimuli in Experiment 1 were identical to the targets in Yankouskaya et al. (2012). Due to copyright reasons, it is not possible to provide an identical illustration of the faces, but Fig. 1 can serve as an example (and see the Appendix from Yankouskaya et al., 2012). The redundant target consisted of Person A with a sad expression. As such, the target identity for the current study was “Person A”, whereas the target emotion was “sad.” For the single-target identity trials, Person A was shown with a happy expression, and for the single-target emotion trials, Person B was presented with a sad expression. Three completely different faces were chosen as nontarget stimuli (i.e., Person C expressing fear, Person C expressing anger, Person D expressing fear; Fig. 1). In contrast to Yankouskaya et al. (2012), no overlap existed between the target and nontarget stimuli for identity nor expression. We hypothesized that linking the nontarget identity “Person B” and nontarget expression “happy” to a “target present”—*and* a “target not present”—response could potentially lead to a response conflict, reflected in longer reaction times on the single-target trials (i.e., happy Person A, and sad Person B), and thus resulting in a false redundancy gain for the redundant target trials. Furthermore, a disadvantage of the redundancy gain paradigm is the multiple presentation of the targets. More specifically, the target identity (i.e., Person A) and the target emotion (i.e., sad) are necessarily presented twice: Once in the redundant target face, and each once in a single-target face. As such, shorter reaction times on the redundant target trials could be due to a familiarity effect. To overcome this issue, we copied the structure of the target trials and created a redundant trial within the non-target trials (i.e., Person C expressing fear), and two single non-target trials (i.e., Person C with an angry expression, and Person D with fear). If the mere multiple presentation of one identity and one expression is the cause of a redundancy gain in the target trials, then a comparable gain for the redundant trial in the nontargets should be apparent.

All stimuli were cropped using Adobe Photoshop and were presented in grayscales on a black background.

#### Design and procedure

All participants were tested individually in a dimly lit room, and were seated approximately 80 cm from the screen, on which the entire experiment was presented. Before the practice run and the actual study took place, participants received elaborate on-screen instructions. They were told that a series of faces with different identities expressing different emotions would be presented, and it was their task to indicate whether the presented face was a picture of Person A, and/or whether the face was expressing a sad emotion, by pressing a target-present key. If none of these aspects was present in the stimulus face, participants were required to press a target-absent key. After these initial instructions, subjects were asked to make a mental image of a sad face, and were then shown two faces, which were not used in the actual study, expressing a sad emotion. Afterwards, Person A with a neutral expression was presented for a fixed amount of time (7 s) for every participant. The last instruction screen reminded the participants that they had to indicate, as fast as possible, whether the presented face was a picture of Person A, and/or a person expressing a sad emotion.

After these instructions, a practice run of 60 trials (i.e., 10 per condition) were presented at random to the participant. For Experiment 1, no feedback was provided to prevent a pictorial matching strategy, since only six original images were used throughout the experiment. When the practice trials were completed, subjects received the instruction to indicate whether the targets were present or not for the final time, after which the actual study commenced. A total of 600 trials were divided into six blocks of 100 trials. As such, 100 trials per condition were shown throughout the experiment. Every trial started with a fixation cross (1° × 1°), presented in the middle of the screen, and was followed by the stimulus face (8.5° × 12.3°) after 500 ms. The face remained on screen until the participant had decided whether the target identity and/or target expression were present or not.

### Results

Before turning to the analyses, it should be noted that not all participants understood the task correctly. In order to obtain 20 data sets, 23 participants had to be tested. In other words, three participants had to be discarded as a result of systematic erroneous judgements (i.e., systematically responding “target present” on nontarget trials, or vice versa, resulting in over 90% errors on certain conditions). Upper and lower bounds to determine outliers were different due to a positively skewed distribution of the reaction times, which was present in all participants. As such, outliers were removed by eliminating reaction times below 250 ms and above the conditional mean plus three conditional standard deviations, for every participant separately, which resulted in the removal of .25% of the total data. It should be noted here that conclusions and interpretation of the data remained unchanged whether outliers were removed or not. This was true for this and all following experiments.

We were specifically interested in the hypothesis that identity and emotion coactivate. To this end, we first conducted a repeated-measures ANOVA on the overall accuracy and correct reaction times. If a redundancy gain is apparent in the data, the accuracy on redundant trials should be higher compared to the two single-target trials for identity and emotion, and correct reaction times should be lower. However, since the independent-race model also predicts overall better scores for the redundant trials, a second analysis was conducted based on the aforementioned inequality to check whether our data fit an independent model or a coactivation model. If the latter is true, the inequality should be violated. To this end, we calculated individual and overall cumulative distribution functions (CDFs) with the MATLAB script provided by Ulrich, Miller, and Schröter (2007) and adapted by Alla Yankouskaya (personal communication). CDFs were constructed based on the correct reaction times of redundant trials (Redundant), and single-target trials, separately (Identity and Emotion) and combined (I + E). The combined CDF for the single-target trials was calculated by taking the 100 fastest trials of the single-target identity and single-target emotion trials. If our data fit a coactivation model, the CDF for the redundant trials should exceed the CDF of the combined single-target trials, thus violating the inequality.

#### rmANOVA

Accuracy and correct reaction times are shown in Table 1. The overall rmANOVA with targets as single factor (redundant, identity, and emotion) revealed a significant main effect for accuracy: *F*(2, 38) = 3.75, *p* = .033, η² = .165, and Bonferroni corrected pairwise comparisons revealed a significant difference between identity (*M* = 99%, *SD* = .019%) and emotion trials (*M* = 97%, *SD* = .026%): *t*(19) = 2.65, *p* = .047, *d* = .59. The analyses for correct reaction times revealed a significant main effect of targets: *F*(2, 38) = 9.16, *p* = .001, η² = .325, and significant differences between redundant (*M* = 741 ms, *SD* = 148 ms) and identity trials (*M* = 690 ms, *SD* = 141 ms): *t*(19) = 3.55, *p* = .006, *d* = .79; and emotion (*M* = 763 ms, *SD* = 140 ms) and identity trials: *t*(19) = 3.93, *p* = .003, *d* = .88, after being corrected with Bonferroni for multiple pairwise comparisons. Based on the rmANOVA we thus cannot conclude that a redundancy gain was apparent in the present Experiment 1. In contrast, results seem to be in line with predictions from a race model, with fastest reaction times on single-target identity trials, and a speeded response (although not significant) when identity and emotion are combined.

**Table 1:** Correct responses (%) and correct reaction times (ms) for Experiments (Exp) 1–6, expressed as mean (standard deviation), for redundant trials (Red), single-target identity trials (Identity), single-target emotion trials (Emotion), Nontarget 1 trials (NT1), Nontarget 2 trials (NT2), and Nontarget 3 trials (NT3)

	Exp	Red	Identity	Emotion	NT1	NT2	NT3
Accuracy	1	98 (.029)	99 (.019)	97 (.026)	98 (.039)	99 (.032)	98 (.026)
	2	93 (.057)	97 (.026)	93 (.056)	97 (.031)	98 (.024)	92 (.050)
	3	98 (.022)	98 (.020)	95 (.040)	99 (.010)	99 (.011)	96 (.042)
	4	95 (.070)	95 (.066)	94 (.040)	98 (.018)	99 (.009)	97 (.041)
	5	96 (.038)	92 (.057)	87 (.082)	90 (.068)	97 (.033)	96 (.031)
	6	94 (.058)	89 (.079)	83 (.108)	88 (.081)	94 (.072)	93 (.071)
cRT	1	741 (148)	690 (141)	763 (140)	746 (169)	741 (152)	895 (245)
	2	608 (78)	567 (68)	623 (92)	578 (60)	589 (72)	685 (90)
	3	596 (44)	580 (57)	664 (91)	618 (58)	615 (75)	682 (85)
	4	650 (142)	637 (105)	643 (95)	653 (103)	627 (118)	698 (133)
	5	564 (92)	604 (108)	619 (107)	661 (129)	612 (106)	609 (106)
	6	593 (106)	619 (88)	637 (105)	688 (113)	658 (109)	661 (100)

#### CDF

**Fig. 2:**
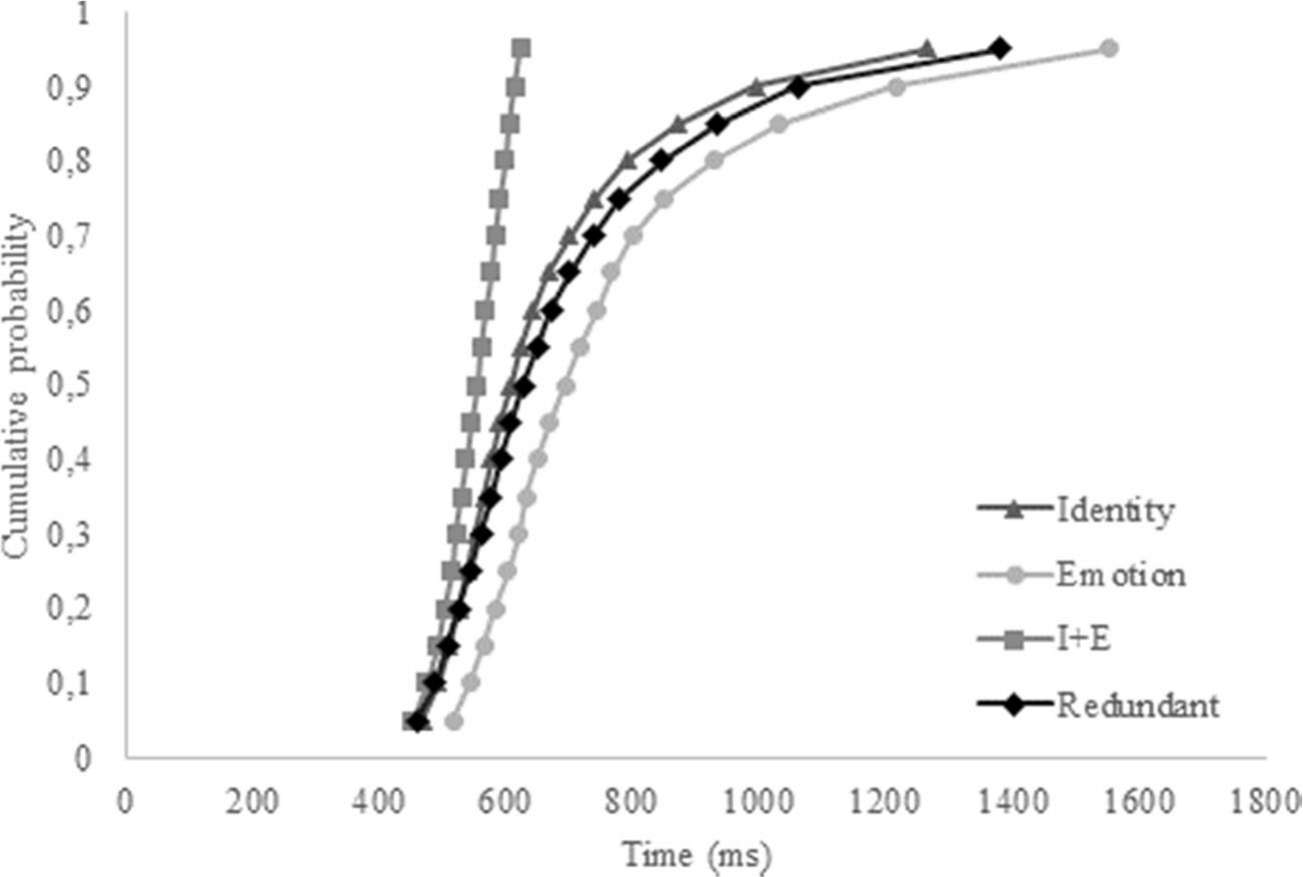
Cumulative probability distribution functions (CDFs) for the single-target identity, single-target emotion, combined single-target trials (I+E), and the redundant trials for Experiment 1

Although no redundancy gain was present in the overall accuracy and correct reaction times, in terms of faster RTs on redundant trials relative to the single-target trials, CDFs were calculated nonetheless, to shed more light on the distribution of the reaction times for the different target-trials. The overall CDF reflects the results from the pairwise comparisons (see Fig. 2). It seems that participants were generally faster on the single-target identity trials, compared to the single-target emotion trials and the redundant trials. The redundant trials did not exceed the combined trials at any point, thus refuting the coactivation model for the current data. Moreover, only four of 20 individual CDFs revealed a redundancy gain.

Because the redundant trials revealed a cost rather than a benefit compared with single-target identity trials, we checked for serial processing. To this aim, we compared the distribution of the redundant trials to the fastest of the single-target trials (i.e., identity), in accordance to the Grice inequality (Grice, Canham, & Gwynne, 1984):

$$ P\left(\mathrm{RT}<t|\ \mathrm{ID}+\mathrm{EMO}\right)\ge \operatorname{MAX}\ \left[P\left(\mathrm{RT}<\mathrm{t}|\ \mathrm{ID}\right),P\left(\mathrm{RT}<t|\ \mathrm{EMO}\right)\right]. $$

This inequality states that the probability of a response RT before a given time *t* on redundant trials (ID + EMO) must be equal to or greater than the fastest of the single-target trials. Evidence for serial processing of identity and emotion is found whenever the inequality is violated. Although a total of 20 percentile points were calculated (i.e., .05, .10, .15, .20, …), we checked the inequality on 10 percentile points, starting from .05, .15, .25, and so forth until .95 to limit the number of paired-samples *t* tests. One-tailed paired *t* tests revealed that the inequality was violated for the .45, .55, .65, .75, and .85 percentiles (*p* < .05), indicative for the serial processing of identity and emotion in Experiment 1.

### Discussion

Results from Experiment 1 point to an independent, serial processing of identity and emotion, as evidenced by accuracy-rates and correct reaction times for redundant trials that do not exceed the single-target trials, as well as CDFs that confirm the inequality of Miller (1982) but violate the inequality of Grice (Grice et al., 1984), at least in the slower part of the distribution. As such, for Experiment 1, the hypothesis of a co-activation, as was found in Yankouskaya et al. (2012), was not confirmed.

Because we believe that the lack of a redundancy gain could be attributable to multiple issues, we will first discuss and manipulate several different factors independently before turning to a perfect replication of Yankouskaya et al. (2012), namely lack of feedback (Experiment 2), stimulus set factors (Experiment 3), and stimulus contingencies (Experiment 4). The decision to implement feedback during the practice trials in Experiment 2 was based on two observations. First, the reaction times from Yankouskaya et al. (2012) for the target conditions are much faster (red: 536 ms, id: 669 ms, emo: 664 ms) compared with ours (red: 741 ms, id: 690 ms, emo: 763 ms). As noted from the CDFs, this was mainly the result of more extreme latencies in our study, presumably increasing the mean reaction times. Second, we tested 23 participants in total before reaching 20 data sets of useable results. Data from three participants had to be discarded since it became apparent that they made inaccurate discriminations between the different conditions, wrongfully indicating “target present” for nontargets, or “target absent” for target trials. We hypothesize that these longer reaction times potentially reflect a deeper cognitive elaboration throughout the study, with participants being more uncertain about the accuracy of their answer, since they never received any confirmation. Relevant to this issue is the finding that the memory bias for faces depicting direct gaze (e.g., Mason, Hood, & Macrae, 2004; Nakashima, Langton, & Yoshikawa, 2012) is affected by task instruction (Daury, 2011). Whereas a simple “old/new” task elicited a bias for faces with direct gaze, a more elaborate “remember/know/guess” task eliminated this effect. This points to the sensitivity of the effect (i.e., interaction identity and gaze perception) and its dependency on task instruction. Seeing that the current hypothesis is somewhat similar (i.e., interaction identity and emotion perception), it is possible that this effect too is dependent on task instruction (here: feedback during the practice trials). As such, in Experiment 2, feedback on performance during the 60 practice trials was implemented.

## Experiment 2

### Method

#### Participants

Twenty new students were selected to take part in Experiment 2 (17 female, three male, range: 17–21 years, *M* = 18.2, *SD* = 0.9). Informed consent procedures were identical to Experiment 1.

#### Stimuli, design, and procedure

Choice of stimuli and procedure for Experiment 2 were completely identical to Experiment 1 (see Fig. 1), with the exception that feedback on accuracy was provided during the 60 practice trials (10 per condition).

### Results

The calculation of outliers, which was identical to Experiment 1, resulted in the removal of 0.18% of the total data. In contrast to Experiment 1, no participants had to be removed from the data set due to misinterpretation of the task.

#### rmANOVA

Accuracy and correct reaction times are shown in Table 1. The first rmANOVA on the accuracy data of targets revealed a significant main effect: *F*(2, 38) = 8.78, *p* = .001, η² = .316 . Further Bonferroni corrected pairwise comparisons point to significant differences between redundant (*M* = 93%, *SD* = .057%) and identity trials (*M* = 97%, *SD* = .026%): *t*(19) = 3.93, *p* = .003, *d* = .88; and between emotion (*M* = 93%, *SD* = .056%) and identity trials: *t*(19) = 3.58, *p* = .006, *d* = .80. Results for correct reaction times reflected these findings, with a significant main effect of targets: *F*(2, 38) = 19.48, *p* < .001, η² = .506, pointing to significant differences in reaction times depending on condition. Pairwise comparisons revealed that these differences stem from redundant (*M* = 608 ms, *SD* = 78 ms) and identity trials (*M* = 567 ms, *SD* = 68 ms): *t*(19) = 4.85, *p* < .001, *d* = 1.08; and emotion (*M* = 623 ms, *SD* = 92 ms) and identity trials: *t*(19) = 5.97, *p* < .001, *d* = 1.33.

#### CDF

**Fig. 3:**
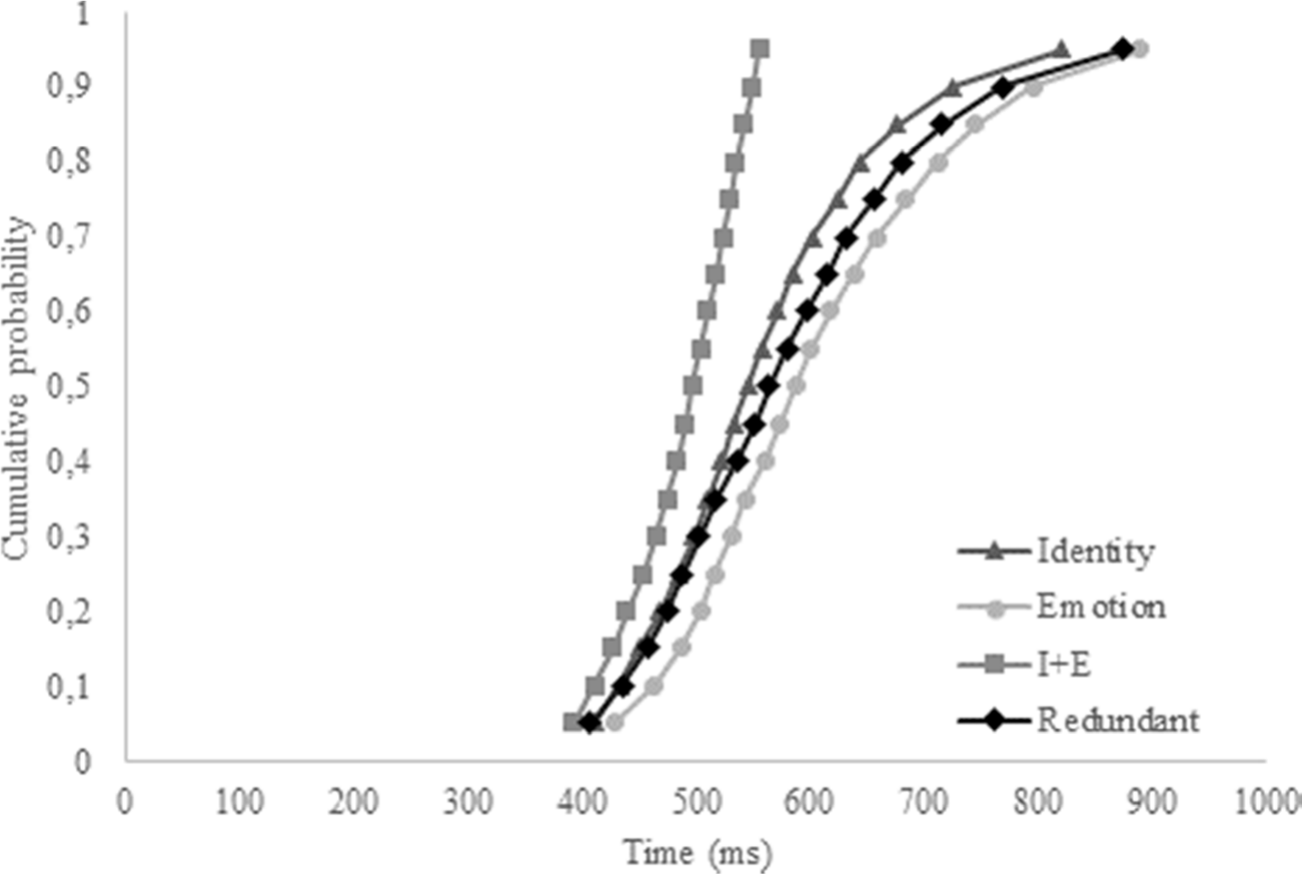
Cumulative probability distribution functions (CDFs) of the single-target identity, single-target emotion, combined single-target trials (I+E), and the redundant trials for Experiment 2

The overall CDF reflected these findings (see Fig. 3). Again, the CDF from the identity trials seems to exceed the CDFs from redundant and emotion trials. Furthermore, relevant to answer the question whether our data fit an independent model or a coactivation model, the CDF from the redundant trials never exceeded the CDF from the combined identity and emotion trials, supporting the independent model. Again, visual inspection of individual CDFs revealed that a minority of participants experienced a redundancy gain (i.e., seven of 20). Instead, one-tailed *t* tests revealed that the Grice inequality (Grice et al., 1984) was violated for the .45, .55, .65, .75, and .85 percentiles (*p* < .05), again indicative for the serial processing of identity and emotion in Experiment 2.

### Discussion

Results were in line with the data from Experiment 1. No evidence of coactive processing of identity and emotion was apparent for Experiment 2. Again, results seem to reveal serial processing for identity and emotion in the slower part of the distribution, with primary processing (i.e., better accuracy and faster reaction time) for the single-target identity stimulus face. Nonetheless, our manipulation (i.e., feedback) did have an effect on the overall reaction times. Participants were, in general, faster to respond “target present” and none of the data had to be discarded due to a misunderstanding of the task. Despite this result, the hypothesis was again not confirmed.

The remarkable finding of higher accuracy and faster reaction times for the single-target identity stimulus face in Experiments 1 and 2 possibly reflects a pop-out effect of this face, potentially facilitating further steps of serial processing. Together with the fact that this is the only stimulus depicting a smile, it is also noteworthy that the target identity (i.e., Person A), which is paired with the smile in the single-target identity trials, is somewhat more plump or chubby compared with the other faces. It is possible that participants quickly learned to respond “target present” whenever the single-target identity face appeared on-screen, because the combination of the smile further enhanced the plump appearance of the face, making it stand out compared to the other stimuli, thus masking a potential redundancy gain. Furthermore, a second possibility states that the happy face stood out compared to the other stimuli because a smile holds a certain degree of “social value” or “power” compared with other emotions (Baudouin, Gilibert, Sansone, & Tiberghien, 2000; Shimamura, Ross, & Bennett, 2006), and the smiling stimulus face was the only face depicting a positive emotion. In order to systematically investigate these hypotheses, in Experiment 3, the same identities and emotions were used, but instead of Person A, Person B was now the target identity, thus removing the chubby appearance from the target identity.

## Experiment 3

### Method

#### Participants

Twenty new undergraduate students completed Experiment 3 (19 female, one male, range: 17–22 years, *M* = 18.1, *SD* = 1). Informed consent procedures were identical to Experiments 1 and 2.

#### Stimuli, design, and procedure

**Fig. 4:**
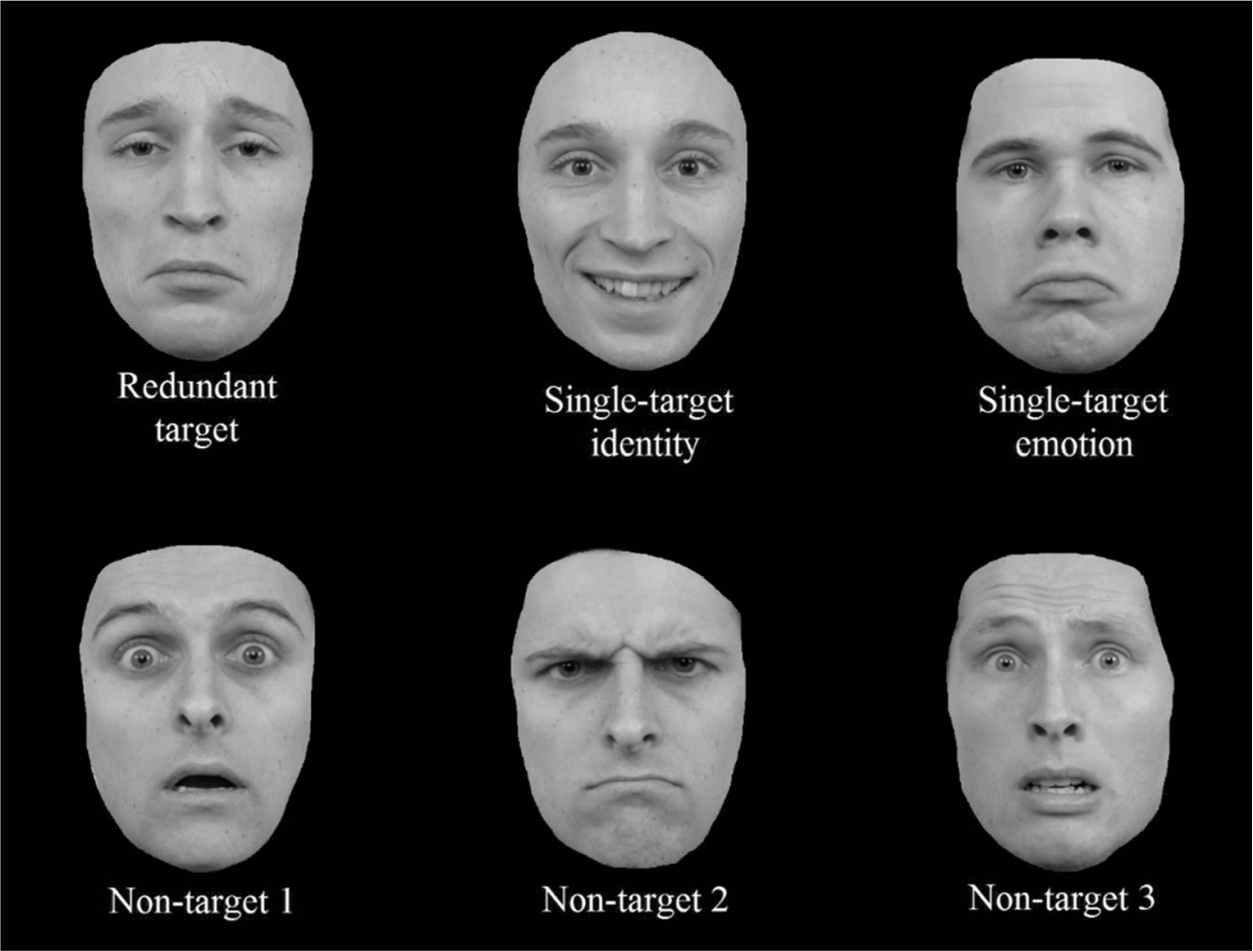
Example faces for Experiment 3, taken from the Radboud face database (Langner et al., 2010). No changes were made to the nontargets, but the identities for the targets were switched. As such, the redundant target now consisted of Person B with a sad expression, the single-target identity was Person B smiling, and the single-target emotion was Person A with a sad expression. Again, these are not the actual stimuli used, due to copyright restrictions

The identities that were used in the redundant and single-target trials were switched. In other words, Person B was now the target identity, and Person A was not (see Fig. 4). The target emotion was still “sad,” and as such, the redundant target was now Person B expressing a sad emotion, the single-target identity trials consisted of Person B with a happy emotion, and the single-target emotion trials were Person A expressing a sad emotion. Please note that, again, due to copyright restrictions, the faces illustrated in Fig. 4 serve as a mere example, but are not the actual pictures used in the experiment (see the Appendix in Yankouskaya et al., 2012).

No changes were made to the nontarget faces. Other than these changes to the stimulus set, the design, and procedure were identical to Experiment 2 (hence, feedback was provided).

### Results

The calculation of outliers, which was identical to previous experiments, resulted in the removal of 0.21% of the total data.

#### rmANOVA

The Mauchly test to test for sphericity in a repeated measures design proved to be significant: *χ*²(2) = 20.50, *p* < .001, and as such, a Greenhouse–Geisser correction (GG = .595) was applied to the degrees of freedom of the *F* statistic. The rmANOVA revealed a significant main effect of targets: *F*(1.19, 22.62) = 13.17, *p* = .001, η² = .409; and the Bonferroni corrected pairwise comparisons revealed significant differences between redundant (*M* = 98%, *SD* = .022%) and emotion trials (*M* = 95%, *SD* = .04%): *t*(19) = 3.86, *p* = .003, *d* = .86; and between identity (*M* = 98%, *SD* = .02%) and emotion trials: *t*(19) = 3.63, *p* = .005, *d* = .81. For correct reaction times, the Mauchly test was again significant: *χ*²(2) = 11.61, *p* = .003, GG = .678. The main effect of targets was also significant: *F*(1.36, 25.76) = 32.75, *p* < .001, η² = .633, and further Bonferroni-corrected pairwise comparisons point to a significant difference between the redundant (*M* = 596 ms, *SD* = 44 ms) and emotion trials (*M* = 664 ms, *SD* = 91 ms): *t*(19) = 4.85, *p* < .001, *d* = 1.08, and between the identity (*M* = 580 ms, *SD* = 57 ms) and emotion trials: *t*(19) = 7.80, *p* < .001, *d* = 1.74.

#### CDF

**Fig. 5:**
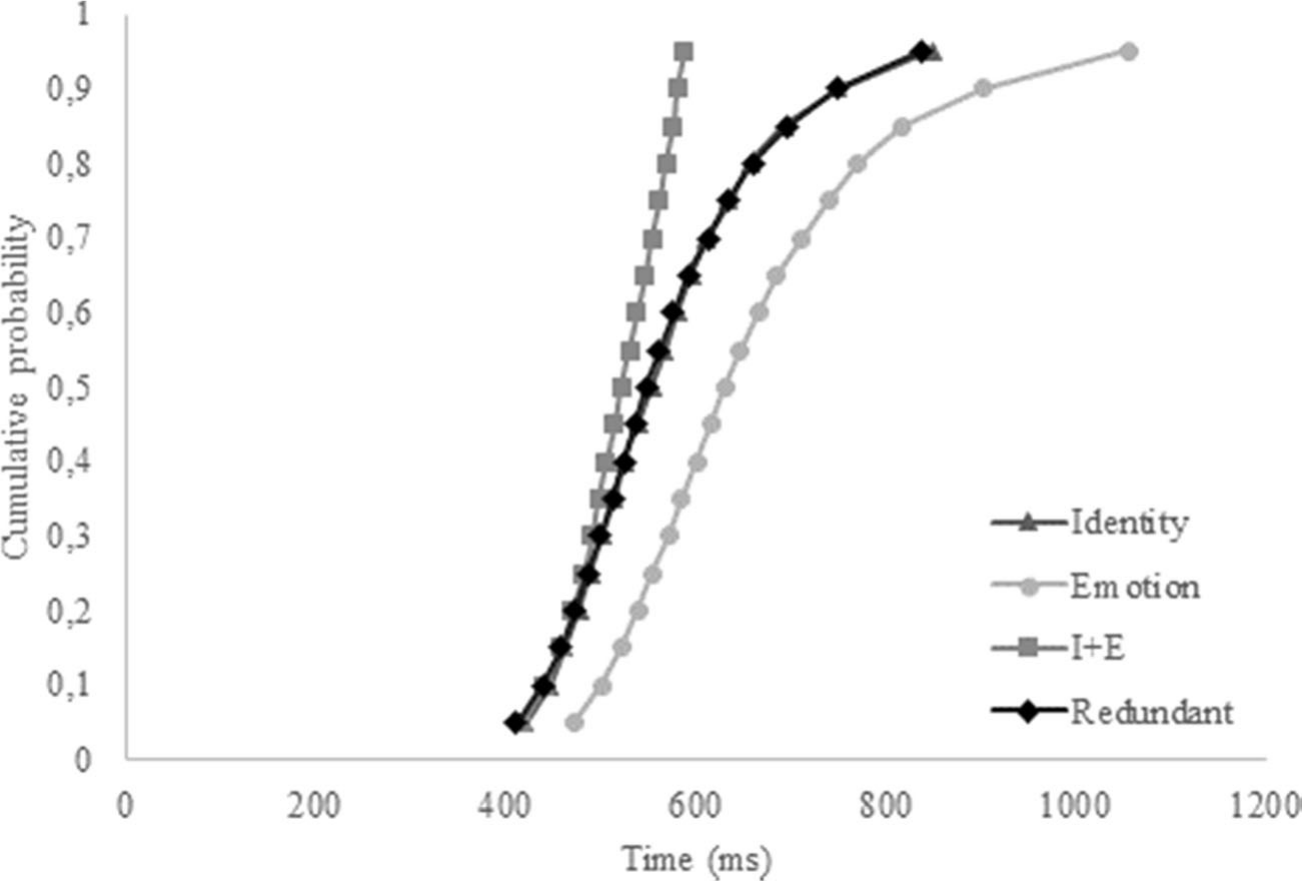
Cumulative probability distribution functions (CDFs) of the single-target identity, single-target emotion, combined single-target trials (I+E), and the redundant trials for Experiment 3

Results from accuracy and correct reaction times do not reflect a coactive processing of identity and emotion (see Fig. 5). Likewise, the CDF of the redundant trials does not exceed the CDF of the combined single-target trials. Furthermore, only five of 20 participants revealed a redundancy gain, as became clear from visual inspection of individual CDFs. Different to previous experiments, the single-target identity trials do not show a “pop-out” anymore, and latencies for redundant trials are now comparable with those of the single-target identity trials, which is reflected by the confirmation of the Grice inequality (Grice et al., 1984).

### Discussion

Switching the target identity, and thus removing a potential stand-out effect of the identity trials, did not elicit a redundancy gain, but successfully eliminated serial processing of identity and emotion. In the next experiment, we again slightly changed the stimulus set to eliminate possible contingencies. As mentioned, the happy expression was always paired with a “target present” response, even though the happy expression itself was not a target. In other words, the appearance of a smiling face was perfectly correlated to a “target present” expression. Furthermore, the happy expression was not only correlated to a “target present” response, but was also always presented with the same identity (i.e., the target identity). Thus, a perfect correlation existed between the happy expression and the target identity. These so-called response-stimulus contingencies (Mordkoff & Yantis, 1991) could lead to significantly speeded responses on these trials, since participants could make use of multiple sources of information to make a “target present” response instead of the mere presentation of the target identity. Identical contingencies exist for the identity of Person B (Experiments 1 and 2), and Person A (Experiment 3). This identity is not a target by itself, but is perfectly correlated to the presentation of the target emotion “sad” in the single-target emotion trials. However, unlike the single-target identity trials, no speeded reaction times were found for the single-target emotion trials. This could potentially point to the special value or power of the happy expression (Baudouin et al., 2000; Shimamura et al., 2006), possibly enhancing conscious awareness of the perfect contingencies for the single-target identity trials.

**Fig. 6:**
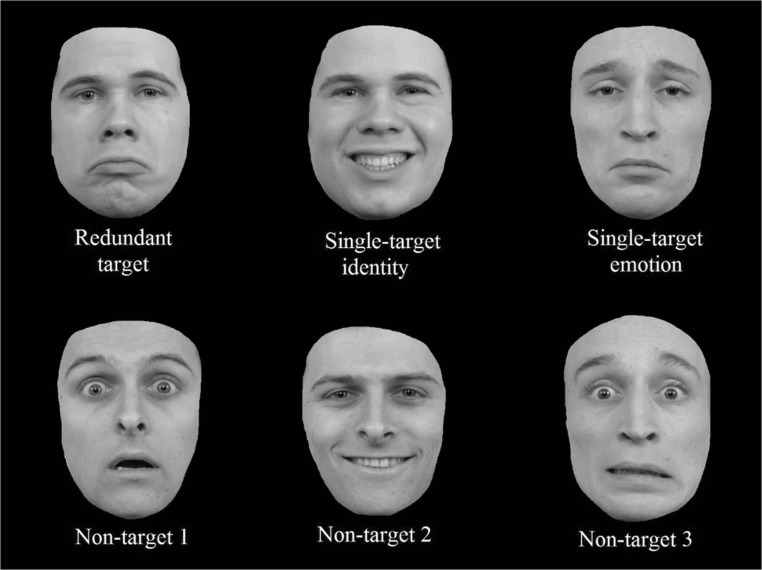
Example of stimuli used in Experiment 4, taken from the Radboud face database (Langner et al., 2010). The targets were identical to Experiments 1 and 2, but the nontarget faces now consisted of Person C expressing fear (Nontarget 1), Person C with a happy expression (Nontarget 2), and Person B expressing fear (Nontarget 3). The actual stimuli are not presented here, due to copyright restrictions

For Experiment 4, the stimulus set was changed to be completely balanced (see Fig. 6). Both the happy expression and the identity of Person B were paired with a target and nontarget, thus eliciting a “target present” response 50% of the time, instead of 100%. The perfect correlation, or contingency, was thus removed, potentially reducing the chance for speeded response times on these trials.

## Experiment 4

### Method

#### Participants

Twenty new undergraduate students took part in Experiment 4 (17 female, three male, range: 18–20 years, *M* = 18.5, *SD* = 0.8). Informed consent procedures were identical to Experiments 1, 2, and 3.

#### Stimuli, design, and procedure

The target faces were the same as those in Experiments 1 and 2 (see Fig. 1; i.e., Person A with a sad expression as redundant target, Person A with a happy expression as single-target identity, and Person B with a sad expression as single-target emotion). However, to create a balanced design, some changes were made to the nontarget stimulus faces. The first nontarget face was still Person C expressing fear, but the second and third non-target face now contained the happy expression (i.e., Person C with a happy expression) and a picture of Person B (i.e., Person B expressing fear), respectively (see Fig. 6). The design and procedure were completely identical to Experiments 2 and 3.

### Results

The calculation of outliers, which was identical to previous experiments, resulted in the removal of .20% of the total data.

#### rmANOVA

None of the analyses of target trials revealed to be significant (*F* < 1).

#### CDF

**Fig. 7:**
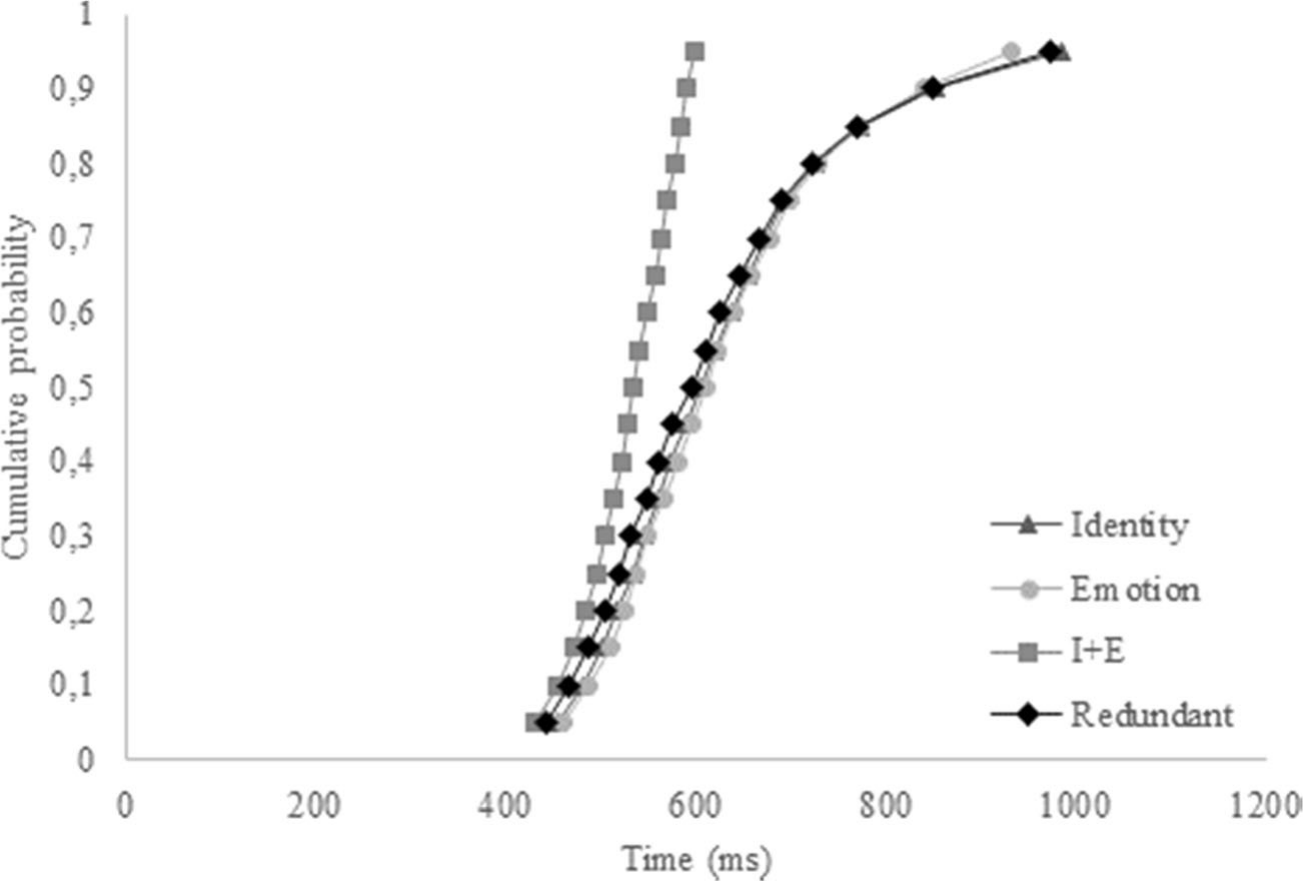
Cumulative probability distribution functions (CDFs) for the single-target identity, single-target emotion, combined single-target trials (I+E), and the redundant trials for Experiment 4

The CDFs of the redundant, identity, and emotion trials are very similar (see Fig. 7). The speeded reaction times for the single-target identity trials are thus eliminated, but there was still no redundancy gain apparent in the data, pointing to independent processing of emotion and identity for Experiment 4. Furthermore, of the twenty participants, only seven revealed a redundancy gain, as became clear from visual inspection of individual CDFs.

### Discussion

The completely balanced design did not elicit a redundancy gain effect. In fact, no differences were found between the target trials, pointing to similar processing of the stimulus faces when contingencies were removed.

Several factors were manipulated that could affect the redundancy gain effect. Whereas none of the manipulations revealed to be successful in eliciting a redundancy gain, removing contingencies within the stimulus set eliminated potential pop-out effects and serial processing of identity and emotion. Although the balanced design from Experiment 4 closely resembles Experiment 1 from Yankouskaya et al. (2012), some factors remain different, and as such, a perfect replication of Yankouskaya et al. (2012) was not yet conducted (see Table 2 for an overview of similarities and differences). For example, although we noticed that several participants experienced difficulties in understanding the task when no feedback was given (Experiment 1), Yankouskaya et al.’s (2012) results were obtained from designs for which participants were not given feedback. As such, in Experiment 5, feedback was again eliminated. Furthermore, we divided the 600 experimental trials in six blocks, whereas this was not done in Yankouskaya et al. (2012). Finally, although they do not mention it explicitly, stimulus presentation times in Yankouskaya et al. (2012) were limited to 2,000 ms, and the target identity was presented to the participants for only 1,500 ms prior to the experiment (information obtained through personal communication).

**Table 2:** Overview of similarities and differences between the first experiment in Yankouskaya et al. (2012) and the six experiments of the current study

	Yankouskaya et al. (2012)	Exp. 1	Exp. 2	Exp. 3	Exp. 4	Exp. 5	Exp. 6
Design							
Target time	1,500 ms	7,000 ms	7,000 ms	7,000 ms	7,000 ms	1,500 ms	1,500 ms
Feedback	No	No	Yes	Yes	Yes	No	No
Breaks	No	Yes	Yes	Yes	Yes	No	No
Stim. set	P1 sad P1 happy P2 sad P2 happy P3 neutr P4 neutr	P1 sad P1 happy P2 sad P4 fear P4 angry P3 fear	P1 sad P1 happy P2 sad P4 fear P4 angry P3 fear	P2 sad P2 happy P1 sad P4 fear P4 angry P3 fear	P1 sad P1 happy P2 sad P4 happy P4 fear P2 fear	P1 sad P1 happy P2 sad P2 happy P3 neutr P4 neutr	P1 sad P1 happy P2 sad P2 happy P3 neutr P4 neutr
Presentation time	2,000 ms	Unlimited	Unlimited	Unlimited	Unlimited	2,000 ms	250 ms
Participants							
Sample size	12	20	20	20	20	20	20
Age	*M* = 23.4 *SD* = 2.1	*M* = 22.9 *SD* = 7.1	*M* = 18.2 *SD* = 0.9	*M* = 18.1 *SD* = 1	*M* = 18.5 *SD* = 0.8	*M* = 18.5 *SD* = 0.9	*M* = 18.5 *SD* = 0.6
Gender	10 ♀ 2 ♂	12 ♀ 8 ♂	17 ♀ 3 ♂	19 ♀ 1 ♂	17 ♀ 3 ♂	20 ♀	20 ♀
Occupation	First year Psychology students	Fourth year Psychology students	First year Psychology students	First year Psychology students	First year Psychology students	First year Psychology students	First year Psychology students
Settings							
Computer	Dell (no other information available)	Dell OptiPlex 7010					
Screen	17-inch (no other information available)	22-inch Vision master pro					
Visual angle	7.1° × 9.3°	8.5° × 12.3°					
Testing conditions	Room with single window, in group (of 3)	Dimly lit, no windows, individual					
Time of testing	Mostly afternoon	Between business hours					

As such, Experiment 5 is a perfect replication of Yankouskaya et al. (2012). More specifically, a few changes were made to Experiment 4: (1) nontargets were changed, (2) no feedback was given, (3) experimental trials were not divided into blocks, (4) the target identity was shown for 1,500 ms instead of 7,000 ms prior to the experiment, and (5) stimuli disappeared from screen after 2,000 ms if no answer was given. All other settings were similar to Yankouskaya et al. (2012) (see Table 2) and thus remained unchanged.

## Experiment 5

### Method

#### Participants

Twenty new undergraduate students took part in Experiment 5 (20 female, range = 18–21 years, *M* = 18.5, *SD* = 0.9). Informed consent procedures were identical to all previous experiments.

#### Stimuli, design, and procedure

**Fig. 8:**
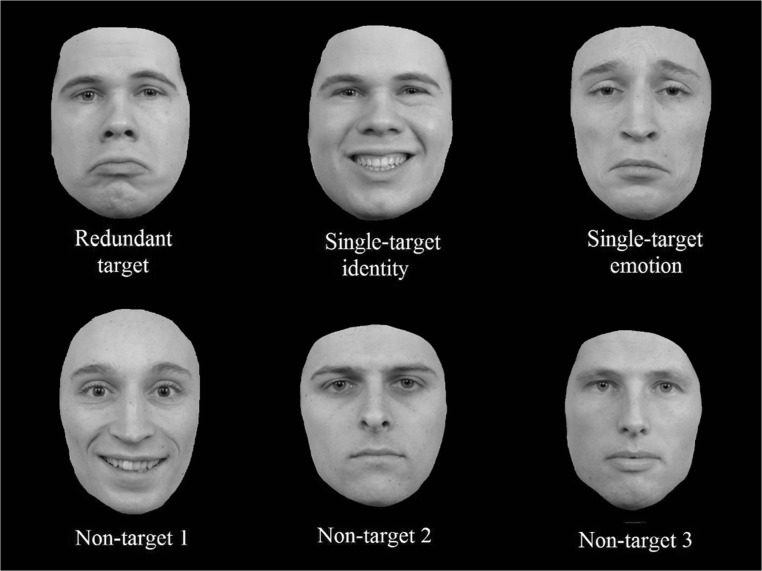
Example of stimuli used in Experiments 5 and 6, taken from the Radboud face database (Langner et al., 2010). The targets were identical to Experiments 1, 2, and 4, but the nontarget faces now consisted of Person B with a happy expression (Nontarget 1), Person C with a neutral expression (Nontarget 2), and Person D with a neutral expression (Nontarget 3). The actual stimuli are not presented here, due to copyright restrictions

The target faces remained the same as in Experiment 1, 2 and 4, but changes were made to the nontargets (see Fig. 8), which now contained a smiling face (same identity as single target emotion stimulus), and two neutral faces, so that the stimulus set was now identical to Experiment 1 of Yankouskaya et al. (2012; see their Appendix). Overall instructions were identical to previous experiments, but as mentioned, participants only saw the target identity (with neutral expression) for 1,500 ms before the experiment. Furthermore, throughout the experiment, stimuli disappeared after 2,000 ms if no answer was given before this time, and no feedback was provided during the practice run. The amount of trials during practice (i.e., 60) and experimental session (i.e., 600) remained unchanged, with a short break in between, but unlike previous experiments, participants were not given a break during the experimental session.

### Results

It should be noted that similar to Experiment 1, not all participants understood the task, as became clear while analyzing the data. A total of 25 participants needed to be tested before reaching a data set of 20 participants with useable data. As such, five participants were discarded from the analyses because of systematic erroneous answers on target trials (i.e., responding “target absent”) or nontarget trials (i.e., responding “target present”). Because no such aberrations were present in Experiments 2–4, we expect that these are caused by the lack of feedback during the practice trials. Calculations of outliers, on the final set of 20 participants, was identical to all previous experiments and resulted in the removal of 0.18% of the total data.

#### rmANOVA

The rmANOVA with Targets as factor (redundant, identity, and emotion) revealed a main effect for accuracy: *F*(2, 38) = 22.07, *p* < .001, η² = .537. Bonferroni-corrected pairwise *t* tests revealed significant differences between redundant (*M* = 96%, *SD* = .038%) and identity trials (*M* = 92%, *SD* = .057%): *t*(19) = 3.98, *p* = .002, *d* = .89; redundant and emotion trials (*M* = 87%, *SD* = .082): *t*(19) = 5.96, *p* < .001, *d* = 1.33; and between identity and emotion trials: *t*(19) = 3.31, *p* = .011, *d* = .74. As such, in contrast to previous experiments, mean accuracy rates point to better processing of the redundant targets compared to the single targets. This was confirmed for correct reaction times, with a main effect of targets: *F*(2, 38) = 20.65, *p* < .001, η² = .521, and significant differences between redundant (*M* = 564 ms, *SD* = 92 ms) and identity trials (*M* = 604 ms, *SD* = 108 ms): *t*(19) = 3.64, *p* = .005, *d* = .81; and redundant and emotion trials (*M* = 619 ms, *SD* = 107 ms): *t*(19) = 7.66, *p* < .001, *d* = 1.71.

#### CDF

**Fig. 9:**
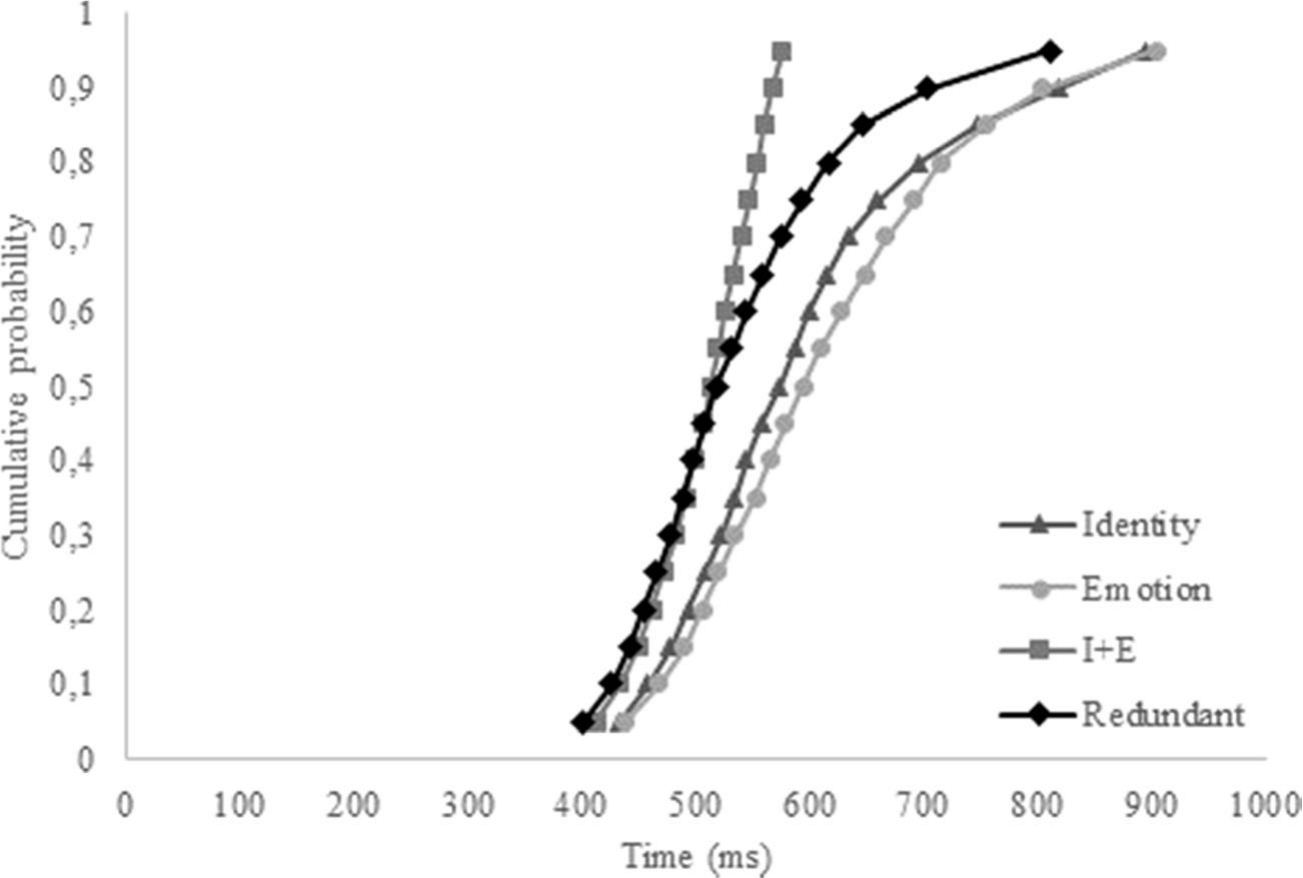
Cumulative probability distribution functions (CDFs) for the single-target identity, single-target emotion, combined single-target trials (I+E), and the redundant trials for Experiment 5

Accuracy and correct reaction time pointed to overall better and faster processing of the redundant targets, and in sharp contrast to previous experiments, the CDF of the redundant targets does not fall completely below the combined CDF of the single target trials (see Fig. 9). As such, one-tailed paired-samples *t* tests were conducted to explore whether the Miller inequality was significantly violated. To reduce Type I error due to multiple comparisons, a limited number of *t* tests were conducted, in accordance with Kiesel, Miller, and Ulrich (2007). We chose to test whether the correct reaction time of redundant targets were significantly faster than the combined single targets at percentiles .05, .10, .15, and .20, as the difference between redundant targets and combined single targets was largest at these points (see Fig. 9). Redundant targets revealed to be processed significantly faster at the .05 percentile: *t*(19) = 2.21, *p* = .020, *d* = .49, and the *t* test reached significance for the .20 percentile: *t*(19) = 1.69, *p* = .054, *d* = .38. As such, the Miller inequality was violated for Experiment 5, indicative for a redundancy gain. This conclusion is further reflected by the individual CDFs. Whereas a minority of participants revealed a redundancy gain in the previous experiments, 15 of 20 participants experienced a redundancy gain for Experiment 5.

### Discussion

Experiment 5 was a perfect replication of the first experiment in Yankouskaya et al. (2012), and indeed revealed a redundancy gain, although only at the fifth percentile. It is difficult to determine which manipulation specifically elicited the redundancy gain, but in accordance to literature on coactivation (e.g., Krummenacher, Müller, & Heller, 2002; Mishler & Neider, 2018; Mordkoff & Danek, 2011) and after personal communication with Alla Yankouskaya, one factor in particular seems most likely to have affected the results. We believe that stimulus presentation time, which was limited to 2,000 ms but was unlimited in all previous experiments, potentially facilitated speeded processing and response. Indeed, overall correct reaction times were lower in Experiment 5 compared with other experiments, and to Experiment 1 in particular (in which feedback was also not provided).

After consideration, which was encouraged by a reviewer’s comment, we believe that unlimited presentation times in Experiments 1–4 might have facilitated multiple saccades and cognitive elaboration, potentially promoting the mental separation of the identity information on the one hand, and the emotion information on the other. Indeed, previous work with redundant targets revealed that redundancy gains were eliminated when the two dimensions were perceived to be originating from different objects (Mordkoff & Danek, 2011), or were further apart (Krummenacher et al., 2002). Although these studies did not adopt faces as stimuli, it is thus possible that for the present experiments, participants treated information from identity and emotion as separate sources when unlimited viewing time was available. Some confirmation for this strategy comes from the evidence of serial processing that was found in Experiments 1 and 2. Relevant to this issue are studies with principle components analyses that reveal only a small overlap between visual features that are used to perceive identity and emotion (i.e., 10%–36%; Calder, 2011; Calder & Young, 2005).

Nonetheless, compared with the redundancy gains that were reported by Yankouskaya et al. (2012), we found a violation of the Miller inequality solely for the .05 percentile in Experiment 5. Following the above mentioned hypothesis that stimulus presentation time might play a crucial role, we limited the presentation to 250 ms in Experiment 6. We predict that further eliminating potential saccades to visual features needed to perceive identity versus emotion potentially promotes integration, as measured by the violation of Miller’s inequality at more percentile-points relative to Experiment 5.

## Experiment 6

### Method

#### Participants

Twenty new undergraduate students took part in Experiment 6 (20 female, range: 18–20 years, *M* = 18.5, *SD* = 0.6). Informed consent procedures were identical to all previous experiments.

#### Stimuli, design, and procedure

Stimuli, design, and procedure were identical to Experiment 5, with the exception that stimulus presentation time was limited to 250 ms.

### Results

A total of 29 participants had to be tested before reaching a set of 20 useable data. We believe that the lack of feedback and short stimulus presentation time caused the errors (i.e., systematically responding “target present” for nontarget trials and “target absent” for target trials) in the nine discarded participants. Elimination of outliers resulted in the removal of .25% of the data.

#### rmANOVA

For accuracy, the rmANOVA with targets revealed a main effect: *F*(2, 38) = 16.57, *p* < .001, η² = .466, and all three Bonferroni-corrected pairwise comparisons proved significant. Performance on redundant (*M* = 94%, *SD* = .058%) was better than performance on identity trials (*M* = 89%, *SD* = .079%): *t*(19) = 2.86, *p* = .030, *d* = .64; and emotion trials (*M* = 83%, *SD* = .108%): *t*(19) = 4.86, *p* < .001, *d* = 1.07. And performance on identity trials was better than on emotion-trials: *t*(19) = 3.54, *p* = .007, *d* = .79. The main effect of Targets was also significant for correct reaction times: *F*(2, 38) = 10.64, *p* < .001, η² = .359, but the only pairwise comparison that proved significant was the difference between redundant (*M* = 593 ms, *SD* = 106 ms) and emotion trials (*M* = 637 ms, *SD* = 105 ms): *t*(19) = 5.57, *p* < .001, *d* = 1.25. No differences were found for the identity trials (*M* = 619 ms, *SD* = 88 ms). Mean accuracy and correct reaction times thus again point to better and faster performance on redundant trials.

#### CDF

**Fig. 10:**
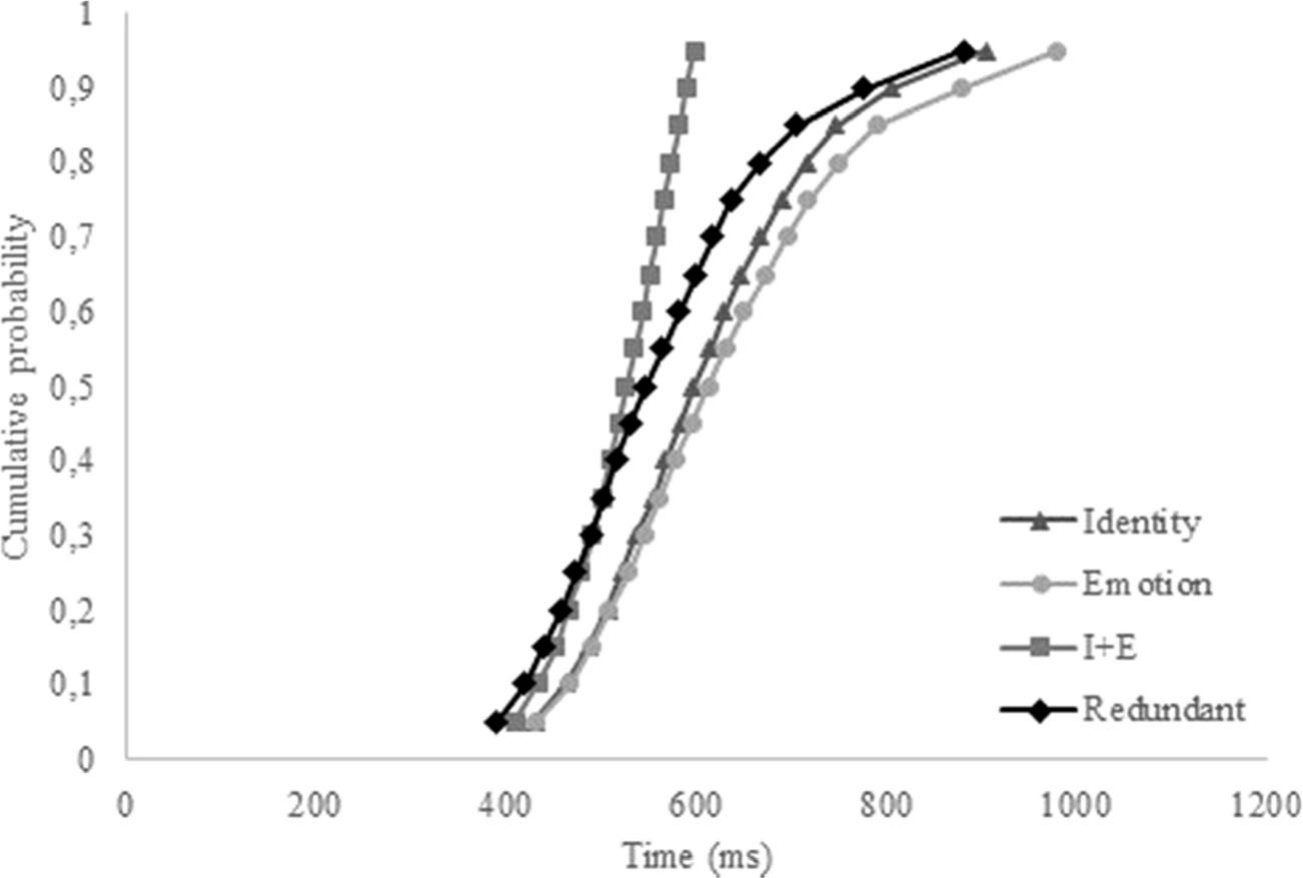
Cumulative probability distribution functions (CDFs) for the single-target identity, single-target emotion, combined single-target trials (I+E), and the redundant trials for Experiment 6

Visual inspection of the CDF seems to confirm faster performance on redundant trials, and similarly to Experiment 5, the CDF of the redundant trials falls before the CDF of the combined single-target trials on lower percentiles (see Fig. 10). One-tailed paired-samples *t* tests were calculated for the .05, .10, .15, and .20 percentiles, but only the .05 revealed a significant difference: *t*(19) = 2.11, *p* = .024, *d* = .47. As such, the Miller inequality was violated at percentile .05, indicative for a redundancy gain for Experiment 6. Visual inspection of the individual CDFs revealed that 13 of 20 participants experienced a redundancy gain.

### Discussion

In contrast with our prediction, further shortening the stimulus presentation time to 250 ms did not increase the number of percentile points on which the Miller inequality was violated. Nonetheless, the Miller inequality was again violated for the .05 percentile, as was the case in Experiment 5, thus confirming the coactive processing for identity and emotion. However, compared with Yankouskaya et al. (2012), who reported a violation of the Miller inequality for the first nine percentiles (i.e., .05, .10, .15, … , .45), the obtained redundancy gains for Experiments 5 and 6 remain limited to the fifth percentile. Nonetheless, the effect sizes of the redundancy gain in Experiments 5 and 6 (i.e., *d =* .49 and *d* = .47) are comparable to the effect sizes reported by Yankouskaya et al. (2012) (i.e., *d* = .60).

It should be noted that by limiting stimulus viewing time to 250 ms we indirectly controlled for multiple saccades, and this hypothesis was thus not tested directly (i.e., by monitoring eye gaze). As such, although restricting stimulus presentation time to 250 ms had no additional effect on the redundancy gain compared with Experiment 5, the hypothesis of multiple saccades as underlying mechanism to explain the lack of redundancy gains for unlimited stimulus presentation (i.e., Experiments 1–4) seems valuable to explore further in the future, given the finding that physical facial features to perceive identity and emotion only show a small overlap (i.e., 10% - 36%; Calder, 2011; Calder & Young, 2005). Furthermore, although very little is known about underlying neural mechanisms, it seems that early visual areas encode both identity and emotion information (Blau et al., 2007; Duchaine and Yovel, 2015; Fox et al., 2009; Rhodes et al., 2015), and it is possible that information bifurcates at later stages in the processing stream (Calder & Young, 2005; Fox et al., 2009).

## General discussion

Our goal for the current experiments was to replicate the results from Yankouskaya et al. (2012), providing evidence for coactive processing of identity and emotion using a divided attention task known as the redundancy gain paradigm. Although the group from Yankouskaya proved to be successful in replicating a redundancy gain for identity and emotion in several studies, with multiple stimulus sets and different populations (Yankouskaya et al., 2012; Yankouskaya et al., 2014a, 2014b; Yankouskaya et al., 2014), we experienced more difficulties to obtain robust redundancy gains in a series of six experiments. Several manipulations to the task instruction and design were applied, and many of them did not reveal a redundancy gain (i.e., providing feedback, changing the stimulus set, eliminating response-stimulus contingencies), although removing response-stimulus contingencies did prove to be successful to eliminate serial processing of identity and emotion. Nonetheless, a perfect replication of Yankouskaya et al. (2012) (Experiment 5) revealed a redundancy gain, which was replicated in Experiment 6. We argued that limiting stimulus presentation time reduces the possibility of multiple saccades and cognitive elaboration, while facilitating speeded processing and rapid integration. In the remainder of the General Discussion, we will discuss several aspects that limit conclusions for the current study and the redundancy gain paradigm in general, and elaborate on factors that can guide future research on face perception with the redundancy gain paradigm.

Although the hypothesized effects of the manipulated factors in the first series of tasks (i.e., Experiments 1–4) were already discussed in the Discussion sections after the experiments, it is important to note that it is difficult to determine their influence on the redundancy gain because these factors were not manipulated in the design that elicited the actual gain (i.e., Experiments 5–6). In other words, it is possible that the obtained redundancy gains are eliminated when feedback and/or stimulus-response contingencies are implemented in the design with limited stimulus presentation time. However, we believe that this is not a likely scenario as Yankouskaya and her colleagues reported redundancy gains in tasks with and without feedback (Yankouskaya et al., 2012; Yankouskaya et al., 2014a, 2014b; Yankouskaya et al., 2014; Yankouskaya, Stolte, Moradi, Rotshtein, & Humphreys, 2017), and contingencies (Yankouskaya et al., 2012). Nonetheless, the fact remains that we did not directly test the influence of these factors on the redundancy gain, and we are thus cautious to conclude that stimulus presentation time is the key factor to elicit a redundancy gain, and believe that future research should study this issue further (see also Mishler & Neider, 2018). A second important aspect that we believe is important to consider in future research on face perception with the redundancy gain paradigm is the selection of stimulus faces and the composition of the stimulus set. Although Yankouskaya et al. (2012) carefully selected pairs of faces with roughly equal discriminability and we used the same stimuli, we acknowledge that not conducting a similar study prior to the actual experiment is a limitation of the current study. Indeed, we observed speeded response times for the single-target identity stimulus in Experiments 1 and 2, which we hypothesized resulted from a pop-out or better discriminability, because the single-target identity was the only smiling stimulus. This possibly led to the violation of the Grice inequality (Grice et al., 1984), indicative for the serial processing of identity and emotion in these experiments. However, the issue of stimulus selection is difficult to resolve given that the redundancy gain paradigm is inherently flawed when it comes to balancing the stimulus factors, as there is a trade-off between response-stimulus contingencies on the one hand and a potential response conflict for single-target trials on the other hand. For example, completely balancing the design, with an equal chance of a “target present” and a “target absent” response for nontarget properties (e.g., “smile” in Experiment 4) might lead to a response conflict on single-target trials, potentially slowing response times on these trials. However, removing the response conflict leads to a correlation of nontarget properties with a “target present” and “target absent” response (e.g., “smile” in Experiments 1–3), which previously has been shown to influence the redundancy gain (Mordkoff & Yantis, 1991).

Several other factors exist that were not manipulated in the present study but that could nonetheless influence the integration between identity and emotion in faces, such as familiarity and perceived self-relevance. For example, there is evidence for a differential effect of familiarity on the processing of facial emotion and identity, with results suggesting an increase of identity and emotion integration for familiar faces compared to unfamiliar faces (Ganel & Goshen-Gottstein, 2004; Yankouskaya et al., 2014a). Furthermore, faster reaction times when naming famous faces depicting a happy expression (Gallegos & Tranel, 2005), and reversely, a heightened feeling of familiarity for faces expressing a positive emotion (Baudouin et al., 2000; Kaufmann & Schweinberger, 2004; but see Johansson, Mecklinger, & Treese, 2004) point to a specific role of familiarity on the processing of positive emotions and vice versa. The current study adopted a divided attention task with *unfamiliar* faces. However, the familiarity of the face possibly affects the quality of processing (e.g., stronger integration between identity and emotion for familiar faces, Ganel & Goshen-Gottstein, 2004; Yankouskaya et al., 2014a), with human observers relying more on specific face features for unfamiliar faces and more on the global/configural information for familiar faces (Bruce, Henderson, Newman, & Burton, 2001; Megreya & Burton, 2006). One could remark that multiple presentation of the face stimuli, as was done in this study, leads to a certain degree of familiarity. However, beside the redundant target, same identities were not usually presented with different emotions, and we believe that the term “familiarity” should be reserved for faces that we encounter regularly, in different formats (e.g., static and in motion, from different viewpoints; Burton & Jenkins, 2011; Johnston & Edmonds, 2009). Studies show that participants are surprisingly bad at matching an image of a static face to a different (but very similar) image of the same face, even when presentation time is unlimited (e.g., Bindemann & Sandford, 2011; Burton & Jenkins, 2011). This difficulty is unlikely to occur with pictures of people with which we are familiar, given our so-called expertise with face recognition. As such, it is not likely that the faces presented in the current study became truly familiar throughout the task. The studies on familiarity thus seem to point to a specifically strong relation between identity and *positive* emotions, but many studies exist reporting enhanced or deeper processing of angry faces (Bach, Schmidt-Daffy, & Dolan, 2014), or fearful faces (Righi et al., 2012). Although a deeper discussion on the precise mechanisms that underlie the outcomes described above falls out of the scope of this paper, these studies provide a common theoretical context to interpret results on interactions between identity and emotion: The social meaning elicited by emotional faces, either negative or positive, influences the degree of perceived self-relevance and potentially results in better face memory and enhanced processing of particularly relevant emotional faces in a given context (i.e., angry colleague at work, smiling partner). Whereas researchers reporting a bias for happy faces argue that a smiling face implicitly expresses approval towards the viewer’s behavior and thus presumably triggers deeper encoding (D’Argembeau, Van der Linden, Comblain, & Etienne, 2003a; Lander & Metcalfe, 2007), others argue that faces with negative expressions denote threat and are thus processed more rapidly and efficiently (Bach et al., 2014; Righi et al., 2012).

In line with this self-relevance hypothesis, newer models of face perception state that face processing is defined by both bottom-up perceptual factors, and top-down social-cognitive factors (Hugenberg, Wilson, See, & Young, 2013; Hugenberg, Young, Bernstein, & Sacco, 2010). The social meaning of emotional expressions presumably affects the processing of identity via top-down mechanisms, a hypothesis that has been confirmed in both behavioral and neural studies (D’Argembeau & Van der Linden, 2007; Godard et al., 2013; Righi et al., 2012). However, if no contextual cues are provided (as with the current study), or no top-down social-cognitive factors are implied, it is difficult to predict which (if any) combinations of identity and emotion are perceived as socially meaningful in the design. To illustrate, the memory bias for happy faces described above was absent in participants suffering from social anxiety, and tipped towards a bias for critical looking faces in this population (Coles & Heimberg, 2005; D’Argembeau, Van der Linden, Etienne, & Comblain, 2003b; Lundh & Öst, 1996; but see Hagemann, Straube, & Schulz, 2016), suggesting that own feelings towards the stimulus-face (i.e., perception of self-relevance) can modulate the direction of an effect.

To summarize and conclude, the focus of the current study revolved around several design-related factors that could influence the redundancy gain effect. We believe that the insights we provided here are useful for future research on redundancy gains in face perception, as only a handful of studies on redundancy gains in faces exist (e.g., Fitousi, 2015) and still very little is known about task-related effects. Indeed, the seemingly robust findings that were reported by Yankouskaya and her colleagues (Yankouskaya et al., 2012; Yankouskaya et al., 2014a, 2014b; Yankouskaya et al., 2014) proved difficult to replicate in the present study (Experiments 1–4). Based on the current results, we advise future researchers to carefully control stimulus discriminability, response-stimulus contingencies, and presentation time. Furthermore, although familiarity and perceived self-relevance were not of interest in the current study, implementation of these concepts with future work on emotion-identity integration seems important. Although an elaborate discussion of this issue falls out of the scope of the current study, the redundancy gain paradigm has limitations with regard to ecological validity and true face perception. More specifically, the limited number of face stimuli, which are presented multiple times, could potentially evoke non-face specific, low-level visual mechanisms, as was the case in Experiments 1 and 2 (i.e., serial processing, potentially facilitated by pop-out properties). As such, researchers should not only keep the question “do emotion and identity integrate” in mind, but also “*why* would emotion and identity integrate,” and adapt the design accordingly. In other words, if it is hypothesized that emotion and identity interact based on perceived self-relevance, for example, then the design should be operationalized in a way that enables manipulation of self-relevance.

Finally, although the initial aim of the current study was to administer the redundancy gain paradigm on a population with congenital prosopagnosia (CP), the lack of significant results prevented this goal and triggered us to instead investigate the design itself. Nonetheless, research on divided attention towards multiple facial aspects in a population with CP could be valuable to build towards a theoretical framework on face perception mechanisms in CP, since a divided attention task reflects real-life face processing much more than selective attention to specific sources of facial information.
